# Bacterial Cellulose Production within a Circular Economy Framework: Utilizing Organic Waste

**DOI:** 10.3390/polym16192735

**Published:** 2024-09-27

**Authors:** Cristina Moreno-Díaz, Salvador González-Arranz, Carmen Martínez-Cerezo

**Affiliations:** Escuela Técnica Superior de Ingeniería y Diseño Industrial, Universidad Politécnica de Madrid, 28012 Madrid, Spain

**Keywords:** bacterial cellulose, extraction methods, biomaterials

## Abstract

Bacterial cellulose (BC) has emerged as a sustainable biomaterial with diverse industrial applications. This paper examines BC production through a circular economy framework, focusing on organic waste as a primary feedstock. It compares static and agitated cultivation methods for BC production, highlighting their advantages and limitations. Static cultivation using Gluconacetobacter xylinum yields high-quality cellulose films but is constrained by lower yields and longer incubation times. Agitated cultivation accelerates production but may affect fiber uniformity. This paper emphasizes sustainability by exploring organic waste materials such as coffee grounds, tea leaves, and food scraps as cost-effective nitrogen and carbon sources. These materials not only lower production costs but also support circular economy principles by converting waste into valuable products. BC produced from these waste sources retains key properties, making it suitable for applications in the textile and other industries. In addition, BC production can align with vegan principles, provided that all additives and processing methods are free of animal-derived components. The paper discusses BC’s potential to replace synthetic fibers in textiles and reduce environmental impact. Case studies show successful BC integration into textile products. In conclusion, this paper calls for more research to optimize BC production processes and explore new industrial applications. Using organic waste in BC production can help industries adopt sustainable practices, reduce environmental footprints, and create high-value materials.

## 1. Introduction

Industrial growth and urban expansion have driven a linear model of production and consumption that has led to the irreversible degradation of ecosystems. This model, based on the extraction, production, and disposal of resources, not only has a negative environmental impact but also presents significant challenges for waste management, especially in large cities [1,2]. In this context, the circular economy emerges as an essential alternative, promoting the reuse of materials and minimization of waste with the aim of achieving sustainable development. Material innovation, particularly biomaterials, plays a crucial role in transitioning to a more circular production system that is less dependent on non-renewable resources.

The widespread use of petroleum-derived plastics has brought significant advances in various technical, industrial, and social areas. However, their non-renewable origin, slow degradation, and the challenges in managing plastic waste are well documented and increasingly concerning issues [3]. It is estimated that only 31 % of plastics are recycled, with a large portion of plastic waste ending up in landfills or oceans, causing significant harm to wildlife and the global economy [4]. In light of these challenges, there is an urgent need to develop sustainable alternatives that not only reduce environmental impact but also integrate into a circular economy model.

**Figure 1:**
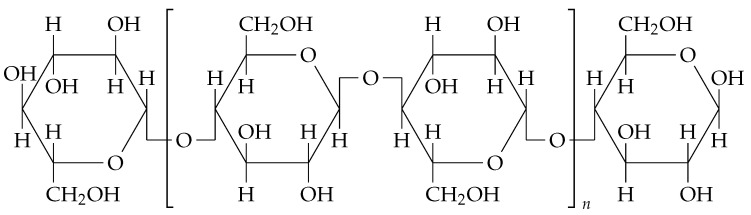
Chemical structure of bacterial cellulose. The structure consists of a non-reducing end, a repeating structural unit (cellobiose), and a reducing end. Adapted from [7].

In response to this need, bacterial cellulose has emerged as a promising biomaterial. Cellulose, the most abundant biopolymer on the planet, is found in a variety of organisms, including plants, fungi, algae, animals, and bacteria [5]. However, cellulose produced by bacteria offers significant advantages over plant-derived cellulose, including higher purity, crystallinity, and better mechanical properties, making it suitable for a wide range of industrial applications [6]. Additionally, BC production from organic waste not only reduces production costs but also supports circular economy principles by converting waste into valuable products. The chemical structure of BC can be seen in Figure 1.

BC cultivation can be performed through static or agitated methods, each with its own advantages and limitations. Static cultivation using *Gluconacetobacter xylinum* produces high-quality cellulose films but is limited by lower yields and longer incubation times. On the other hand, agitated cultivation accelerates production, although it may compromise fiber uniformity [8]. This work compares both cultivation methods, evaluating their viability based on the quality of the material produced and its suitability for specific applications, such as the textile industry.

The textile sector is one of the most polluting industries and is a prime target for significant changes in the materials it uses. Synthetic textiles, mostly derived from petrochemical sources, pose a growing problem due to their low biodegradability and the microplastics they release during use and decomposition [9]. BC, with its exceptional properties, has the potential to replace synthetic fibers in textiles, helping to reduce the environmental footprint of the industry. Moreover, the biodegradability of BC and its potential for composting at the end of its lifecycle make it a sustainable alternative compatible with the circular economy.

In the pursuit of sustainable alternatives to traditional materials, it is important to consider not only the technical and economic viability but also the ethical aspects that guide material choices. Specifically, the use of animal-derived dyes and additives can compromise the vegan nature of products made from bacterial cellulose. While bacterial cellulose is inherently a plant-based and cruelty-free material, its treatment and finishing with certain animal-derived products, such as beeswax or cochineal (used as a dye), could contradict vegan principles.

Therefore, when designing and producing bacterial cellulose-based materials for applications in the textile industry or elsewhere, it is crucial to carefully evaluate and select components and treatments to maintain the vegan integrity of the final product. This not only broadens the appeal of bacterial cellulose as a substitute for conventional materials but also reinforces its role in creating truly sustainable and ethically responsible solutions.

Throughout this study, the potential of BC produced from organic waste, such as coffee grounds, tea leaves, and food scraps, is explored as a viable solution for replacing synthetic fibers in textiles. Case studies demonstrating the successful integration of BC into textile products are presented, highlighting its commercial viability and contribution to reducing environmental impact. Additionally, the importance of continued research and the optimization of BC production processes is emphasized to expand its industrial applications and improve its competitiveness against traditional materials.

## 2. Materials and Methods

In this section, the materials and methods used in this study to produce bacterial cellulose using organic waste as the primary feedstock are outlined. This study focuses on the sustainable production of BC by converting waste materials into valuable resources, thereby supporting the principles of a circular economy.

### 2.1. Cultivation Methods

Studies were carried out with two culture methods: static and agitated. With static culture, the aim was to produce high-quality cellulose films directly, while agitated culture aimed to speed up production.

The first method, static cultivation, is the most common for producing BC. In this case, the mixture of components was left to rest for five to ten days, depending on the desired thickness, in rectangular containers under controlled conditions. During this time, the bacteria consumes oxygen and nutrients, forming a cellulose film on the surface of the medium. This method produces BC sheets with an interconnected 3D structure, making it ideal for biomedical and textile applications. However, it has limitations such as low yield and long incubation times, which complicate its scalability for industrial production. Improvements have been proposed, such as an intermittent nutrient supply method that can significantly increase the thickness of the produced membrane and allow for its collection as it forms. Recent studies have explored the characteristics of this method and ways to increase its efficiency, such as [10].

On the other hand, agitated cultivation is also performed in a controlled environment with an agitator that keeps the cells in constant motion (150 rpm), resulting in faster BC production, typically within a day or a day and a half. This method produces BC in the form of small pellets or aggregates, which results in less cohesion between the fibers and fewer parallel planes. Although the production speed is higher and the oxygen increases the bacterial population and, consequently, the production of BC, an excess of oxygen can reduce the process efficiency. Additionally, if the BC is intended for the textile industry, where having cellulose sheets is more practical, additional steps are required, similar to paper production. Alternatively, to form complex designs, the BC can be extruded manually or through 3D printing, allowing for various shapes and textures to be obtained.

In addition, two ways of creating the initiator medium were compared by adding bacteria. The first is by crushing a SCOBY (Symbiotic Culture of Bacteria and Yeast) with the rest of the culture medium, and the second is by introducing the solution from the previous fermentation, which already contains all the necessary components for the generation of a new BC sheet. The BC sheets obtained were similar, and this second option is more sustainable, as it uses waste from part of the process.

The comparison between materials obtained through different cultivation processes was conducted using the basic recipe without altering the organic source with residues. This ensures a consistent baseline, highlighting the differences attributed to the cultivation techniques themselves rather than variations in the raw material.

### 2.2. Feedstock Preparation

Bacterial cellulose forms as a film on the surface of acetic fermentations and is an extracellular biopolymer free of lignin and hemicellulose. By creating a suitable growth medium for Acetobacter xylinum, BC can be produced with desirable characteristics such as a high water retention capacity, biodegradability, biocompatibility, strong mechanical properties, high thermal stability, a high degree of crystallinity, and it is both non-toxic and non-allergenic.

The pioneering study by Schramm and Hestrim [11] on the production of BC using Acetobacter xylinum in static culture led to the development of a cultivation medium known as H-S, which remains widely used in current research. The key parameters include the cultivation method, carbon and nitrogen sources, as well as the pH and temperature of the medium [12]. The efficiency in BC production largely depends on the bacteria’s ability to synthesize glucose from the carbon substrate, followed by its polymerization into cellulose. Various studies have shown that the optimal pH for BC production ranges from 4.5 to 7.5, with the greatest efficiency around 5, while the optimal temperature is 28 °C [13,14].

Additionally, different carbon and nitrogen sources have been evaluated to maximize BC production. Studies have shown that the use of hydrolyzed molasses can increase BC production by up to 88% compared to glucose, due to the additional micronutrients present [15]. More recently, complex carbon sources derived from by-products or waste from the agricultural industry are being explored [16].

This leads to the use of alternative and more accessible carbon and nitrogen sources. In the present study, these were divided into two groups. On one hand, a basic recipe was used with more accessible carbon and nitrogen sources like sugar or tea obtained from [17], and on the other, organic waste such as coffee grounds, tea leaves, and spoiled fruits and vegetables were employed, substituting some of the standard ingredients. Table 1 presents the proportions of all components used in this study, comparing the basic recipes with those that employed organic waste as alternative sources.

Additionally, it has been demonstrated how these parameters influence BC production and how they evolve over time [18]. Key aspects of the process include the careful adjustment of initial glucose concentrations and additives to maintain the necessary acidic conditions, as well as meticulous cultivation practices to prevent mold growth on the surface. In this study, to ensure that the carbon content was maintained, Brix degrees were measured using refractometry. After comparing the results with other cultivation recipes, it was concluded that with 300 g of organic waste, approximately 5 °Bx should be achieved to ensure good growth and comparable properties to those of other cultures. If this value is not reached, it is necessary to add more organic material. As noted, maintaining an appropriate temperature is essential for the optimal development of the BC layer, ensuring that the produced material reaches the desired thickness.

**Figure 2:**
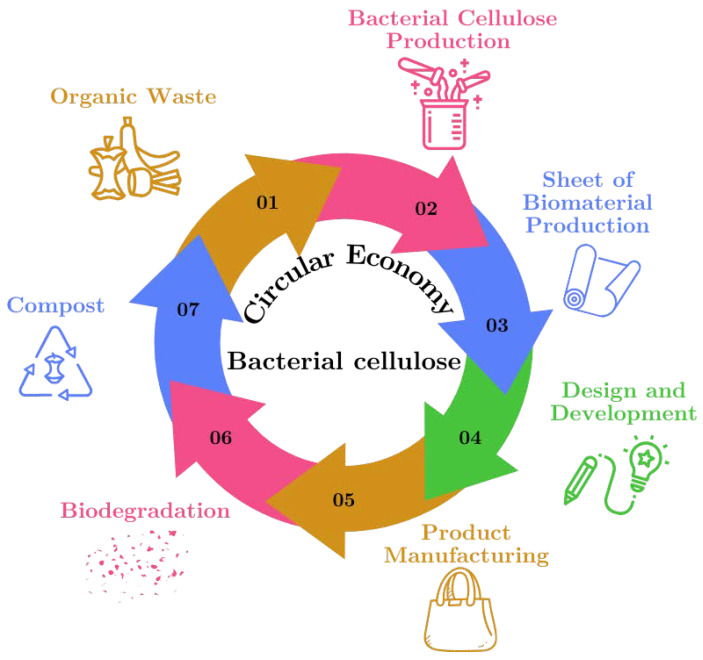
Circular diagram of the sustainable cycle of bacterial cellulose production.

The process of material production within the framework of the “circular economy” using raw materials derived from organic waste is illustrated in Figure 2. This figure shows the sustainable cycle of BC production from organic waste, highlighting how each step of the cycle contributes to reuse and waste reduction.

1. Organic Waste: The cycle begins with the collection of organic waste, such as food scraps, which serve as the primary raw material for BC production.
2. Bacterial Cellulose Production: The organic waste is processed and used to cultivate bacterial cellulose, a versatile biomaterial, in a controlled environment.
3. Biomaterial Sheet Production: The bacterial cellulose is harvested and dried to form biomaterial sheets, which can be used in various applications.
4. Design and Development: These cellulose sheets are then designed and developed into products, highlighting their potential as sustainable alternatives to traditional materials.
5. Product Manufacturing: The designed products are manufactured, utilizing bacterial cellulose to create items such as textiles, packaging, or other consumer goods.
6. Biodegradation: After their useful life, products made from bacterial cellulose undergo biodegradation, breaking down naturally in the environment.
7. Compost: The biodegraded material contributes to compost, returning valuable nutrients to the soil and completing the cycle, which begins anew with the generation of more organic waste.

**Figure 3:**
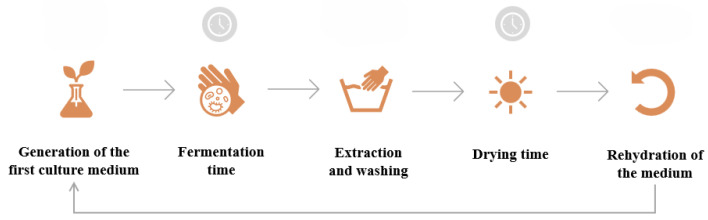
Simplified schematic of bacterial cellulose production with medium reuse. Prepared by the author.

A simplified schematic of bacterial cellulose production is presented in Figure 3, and shows the steps to follow considering the reuse of the medium on which the BC layer grows [19].

### 2.3. Experimental Process for Obtaining BC

**Figure 4:**
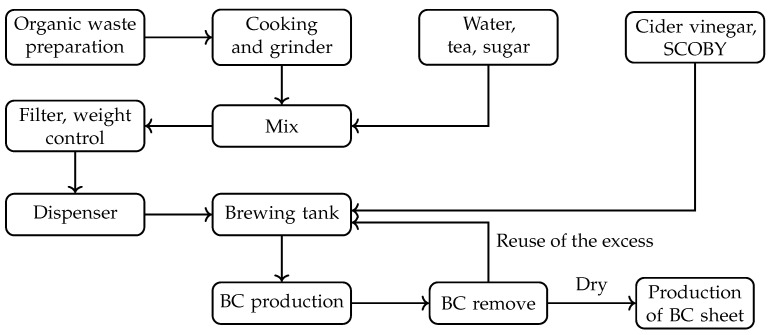
Flowchart of BC production process. Own preparation.

The process of bacterial cellulose production has been described by the authors in both [20], in a “Do it Yourself” (DIY) approach, detailing the collection from local businesses and the production of the material in university laboratories, and in [21], where the production of the biomaterial was simulated on an industrial scale using organic waste from a nearby food company. In all studies, raw materials such as coffee grounds and tea leaves are dried if they are not going to be used immediately to prevent mold growth. For organic waste like fruits and vegetables, especially those obtained from local collection points, an initial cleaning is performed to remove parts that have mold or are already decomposing. Depending on the intended use, the remaining waste is either mixed and ground or pre-sorted by type. The standard production processes is summarized in Figure 4. In this diagram, the specific parts dedicated to dyeing, texture creation, etc., are not included; these will be detailed in the Results Section.

### 2.4. Treatments to Textile Industry

**Figure 5:**
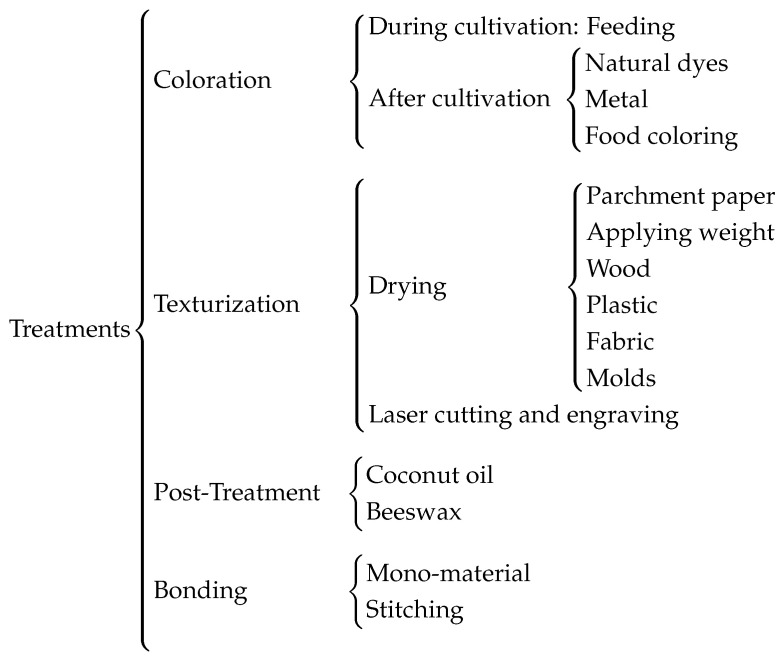
Treatment overview.

With the aim of maintaining sustainability and minimizing environmental impact, various tests were conducted to modify the properties of bacterial cellulose sheets. The targeted changes focused on textile-relevant characteristics such as color and texture. The results of these tests are shown in Figure 5.

The coloring methods applied to the samples are categorized into two groups: those where the cellulose was initially cultivated with pigmented organic matter, and those where colorants were applied after cultivation but before drying.

The texture and surface finish of the samples largely depend on the drying process. This process involves not only the use of molds to achieve specific patterns but also the type of material on which the cellulose is dried, such as smooth plastic, vegetable parchment, or wood. The surface of the drying material significantly influences the cellulose, which tends to adopt the shape, finish, or patterns of the contact material. Additionally, the possibility of creating patterns or printing characters using laser engraving has been evaluated.

Furthermore, treatments were applied to enhance the elasticity or waterproofing of bacterial cellulose while maintaining its biodegradability. Specifically, coconut oil and beeswax were used after drying.

Finally, a key principle of eco-design and sustainability in product development is that products should be made from a single material to facilitate recycling. Therefore, different methods of joining cellulose samples were tested, taking advantage of their ability to fuse during drying. In cases where this was not feasible, stitching between samples using threads of the same cellulose was also explored. Additionally, laser cutting tests were conducted to optimize time and material use.

## 3. Results and Discussion

### 3.1. Comparison of Cultivation Methods

The mechanical tests performed on the bacterial cellulose samples obtained through static and agitated cultivation methods revealed consistent results in terms of tensile strength, elastic modulus, and elongation. Additionally, the influence of adding fermentation liquid from previous processes was evaluated. The values obtained were similar in all cases, suggesting that both methods can produce BC with comparable mechanical properties.

**Figure 6:**
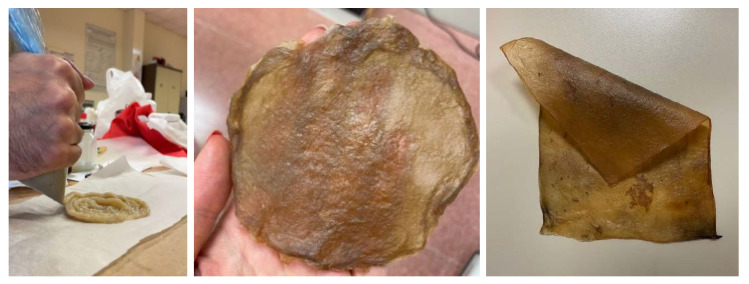
Example of BC extrusion obtained through agitated cultivation for the formation of sheets.

However, all samples produced using the agitated method showed a slight decrease in all mechanical properties compared to those obtained through static cultivation. This difference could be attributed to the lower cohesion between the fibers and the less organized structure of the sheets produced by agitation, as the material obtained through agitation requires additional crushing and extrusion processes to form sheets, which could affect its mechanical strength (see Figure 6).

Table 2 below summarizes the experimental values obtained for each of the mechanical properties evaluated across the different cultivation methods in sheets of approximately 1 mm thickness and manufactured with the basic recipe.

These results confirm that, although the agitated method allows for faster BC production, the mechanical quality of the resulting material is slightly inferior compared to that obtained using the static method. This decrease in quality may be related to the post-processing required to manufacture sheets, which also increases both the time and costs of this method, making it less viable. Thus, it is confirmed that the production of BC sheets is more efficient using the static method, and that adding liquid from previous fermentations does not affect the material’s quality, contributing to reduced production costs.

### 3.2. Utilization of Organic Waste

The use of organic waste materials as feedstocks proved to be cost-effective and aligned with circular economy principles. The BC produced, consistently via static cultivation, retained essential properties such as high water retention, biocompatibility, and biodegradability. However, as described in Cristina et al., the mechanical properties of BC can vary slightly depending on the feedstock used. The sheets obtained using the basic recipe exhibit the best mechanical properties, while samples fed with organic waste show slightly higher rigidity but remain similar overall, Table 3.

**Figure 7:**
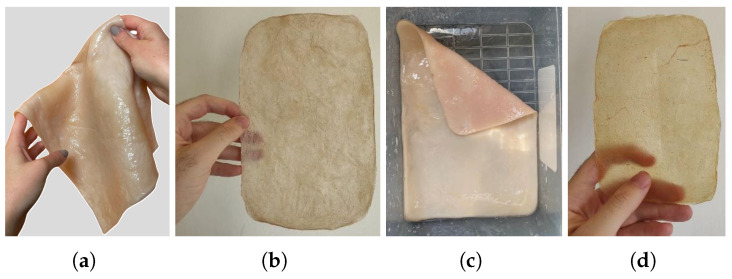
Imagesof the material sheets obtained with different feedstocks: basic (**a**) wet and (**b**) after drying and mixture of organic waste (fruits and vegetables) (**c**) wet and (**d**) after drying.

The appearance of sheets, both before and after drying, is very similar whether they are produced using the basic recipe or a mixture of organic waste (fruit and vegetables) (see Figure 7). Both types exhibit a neutral color and a generally uniform surface.

**Figure 8:**
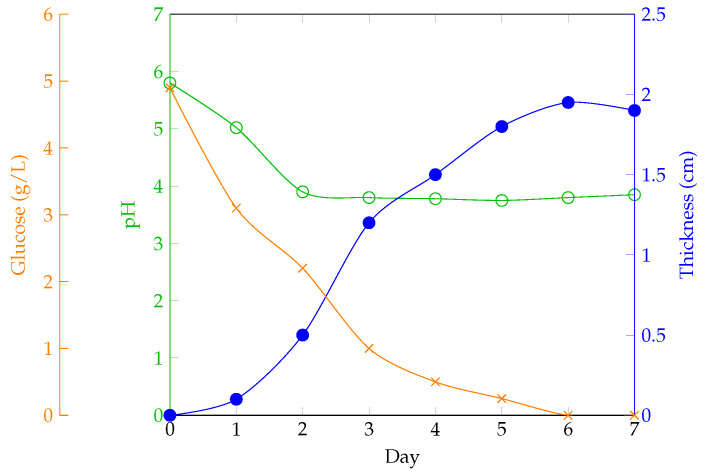
Growth of BC in static culture and variation in its properties and components over time, adapted from [18].

To cultivate BC using fruits or vegetables, whether as waste or fresh materials, it is recommended to choose those rich in sugars (such as sweet fruits, beets, molasses, or potatoes) as they will enhance the cultivation process. The general procedure for using these alternative materials is as follows:

1. Cleaning: Wash the fruit or vegetable scraps with water or a cleaning solution to remove surface dirt and any decomposing residues.
2. Material Preparation: Use a proportion of 300 to 400 g of solid material per liter of water (30% by mass).
3. Sterilization: Boil the water to sterilize the material (100 °C for 30 min). If needed, infuse the selected nitrogen source, 3 g.
4. Grinding: Grind the material together with the water.
5. Filtration: Filter the mixture to retain the liquid, as floating solid particles can hinder cellulose growth on the surface of the medium.
6. Sugar Content Adjustment: Use a Brix meter to ensure the sugar content is around 5 °Bx. Add additional carbon source if necessary.
7. Cooling: Pour the liquid into a sanitized container and cool to 30 °C.
8. Inoculation: Add the SCOBY mother culture or liquid from a previous fermentation and/or apple cider vinegar to make up one-sixth of the total liquid volume.
9. Fermentation: Allow fermentation to proceed for 1 to 5 weeks, depending on the desired final thickness, Figure 8.

Once the cellulose layer has reached the desired thickness (usually 1 cm after three to four weeks), it can be removed from the culture medium, and a new layer can be grown from the same culture. The sheet must then be processed to remove bacterial activity and mitigate the vinegar-like odor by boiling the sheet in water. This cleaning method results in a nearly colorless and inert wet sheet that will regain its color once dry. The sheet is then dehydrated through the drying process for use in products.

### 3.3. Applications in the Textile Industry

The potential of bacterial cellulose to replace synthetic fibers in the textile industry was demonstrated through various case studies, where the integration of BC into textile products showed promising results in terms of sustainability and reduced environmental impact. To further explore this potential, the different valid samples obtained during the experimental process were analyzed and discussed, focusing on the various treatments applied: coloring, textures, bonding, and post-treatment methods to enhance their properties. Whenever possible, these treatments are also carried out using organic waste or by utilizing materials or techniques that do not interfere with the biodegradability of the material. The objective is to identify the optimal way to produce and treat bacterial cellulose for its application in the textile industry, being mindful of its strengths as well as its limitations compared to the materials currently used in the sector.

#### 3.3.1. Coloring Treatments

**Figure 9:**
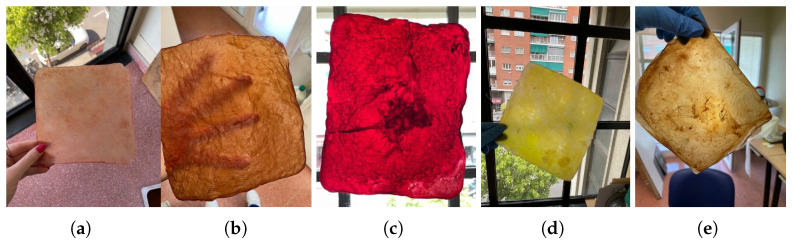
Images of the final appearance of samples dyed during cultivation with (**a**) strawberries, (**b**) beer, (**c**) red wine, (**d**) turmeric, and (**e**) coffee grounds.

**Figure 10:**
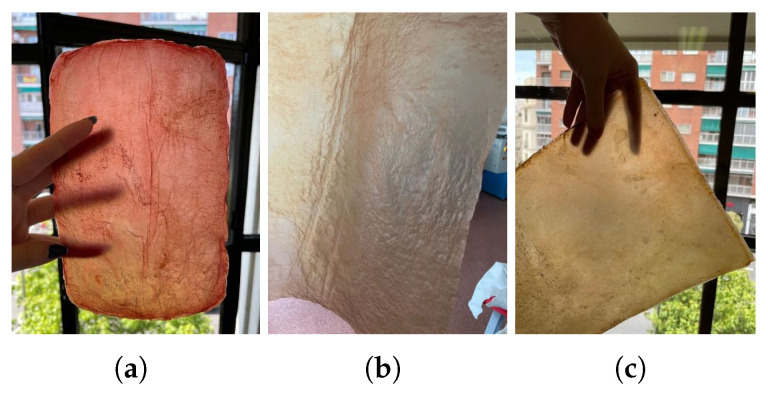
Images of the final appearance of samples dyed with natural dyes such as (**a**) beetroot, (**b**) cochineal, and (**c**) chestnut dye.

**Figure 11:**
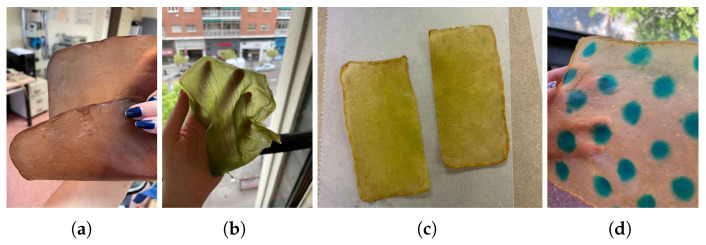
Images of the final appearance of samples: (**a**) after drying between steel sheets, and dyed with food colorings: (**b**) green, (**c**) yellow, and (**d**) blue spotted pattern.

The color of the material is crucial in the textile industry, and there are two main methods of dyeing BC:

1. During cultivation: The color of the material can be influenced by selecting or by adding a greater amount of a specific source or substance, such as strawberries, bananas, or beer. Also, it can be influenced by adding other elements like turmeric or coffee grounds, which also serve as a nitrogen source, as shown in Figure 9.
2. After cultivation: Once the material is formed using a mixture of organic residues, resulting in a neutral color, but before the drying stage, three different tests were conducted:
  (a) Natural dyes: Fruits or vegetables are boiled for one or two hours until the water is infused with their color. Then, the cellulose samples are submerged in this liquid for a day. If pre-extracted dyes are used, boiling is unnecessary; the extract is simply diluted in water. For this study, tests were conducted with beetroot, blackberries, avocado, cochineal, and chestnut dye, Figure 10. The use of cochineal as a red dye should be considered if the goal is to produce a completely vegan substitute for other animal-derived products, especially leather.
  (b) Drying on metal: Samples are placed between steel sheets, separated by a sheet of parchment paper to avoid direct contact. This process, which could also be categorized under texturizing, provides a very smooth finish but significantly darkens the sample. It is crucial to carefully adjust the contact time to avoid excessive oxidation, which could damage the material, Figure 11a.
  (c) Food coloring: Food colorings can be applied in two ways: either by immersing the sample in a solution of the desired coloring or, if the dye is more viscous, by creating patterns with an applicator or brush, Figure 11.

#### 3.3.2. Texturization

The texture and surface finish of the samples largely depend on the drying process used. This process involves the use of not only molds to achieve a specific pattern but also the type of material on which the drying takes place. Whether it is smooth plastic, parchment paper, wood, or other materials, each one significantly affects the final finish of the cellulose, which readily adopts the shape, texture, or imprints of the surface it contacts, as previously discussed.

**Figure 12:**
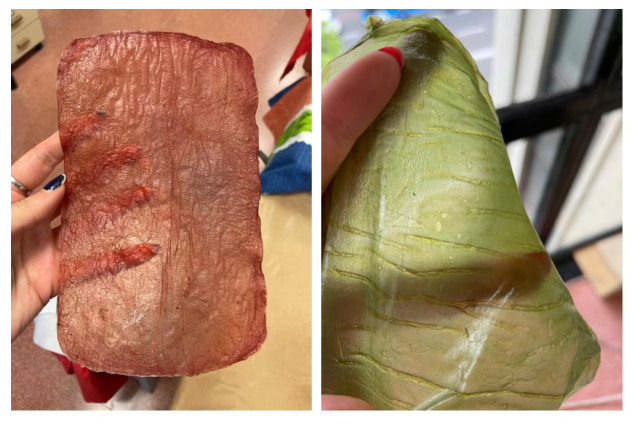
Images of the final appearance of samples dried on parchment paper.

**Figure 13:**
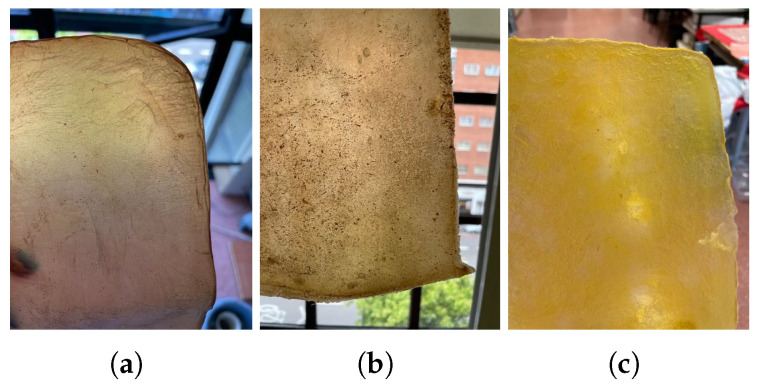
Image of the final appearance of sample dried with weight application (**a**), final appearance of the samples dried directly on wood (**b**) and after the application of beeswax (**c**).

**Figure 14:**
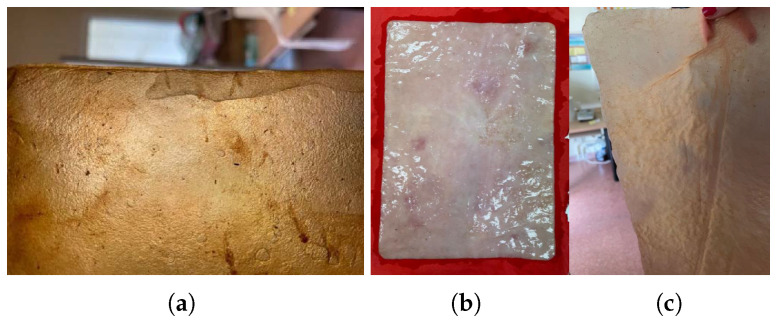
Images of the placement and final appearance of one of the samples dried on plastic (**a**) and on fabric (**b**,**c**).

**Figure 15:**
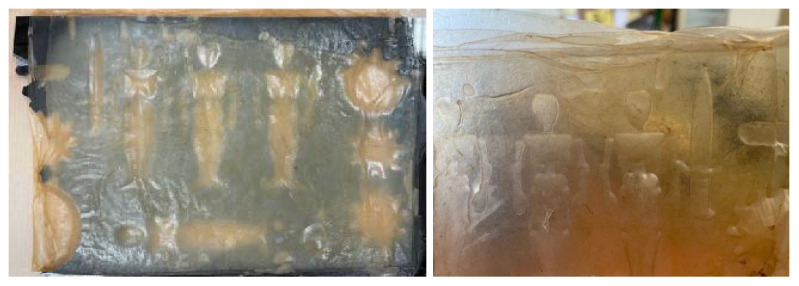
Images of the placement and final appearance of one of the samples dried on a mold.

1. Drying on Parchment Paper: When a freshly harvested bacterial cellulose sample is placed on parchment paper, the initially smooth surface of the paper becomes wrinkled due to the high water content of the cellulose. This wrinkling creates a textured pattern that is transferred onto the cellulose. The extent to which the sample adopts this texture depends on its thickness. Thin samples tend to replicate the texture across the entire material, even deforming slightly if they are only a few millimeters thick when wet. In contrast, thicker samples (around 1 cm) will only display the parchment paper’s pattern on the side that was in contact with the paper during drying, Figure 12.
2. Drying with Weight Application: As mentioned in the section on “*Drying on metal*”, applying weight results in a smoother surface and a completely flat sample. The added pressure ensures that the cellulose dries evenly, eliminating any wrinkles or distortions and achieving a uniform, polished finish, Figure 13.
3. Drying on wood results in a smooth and uniformly surfaced material. However, the strong adhesion of the sample to the wood can lead to difficulties in removing it, potentially causing damage or leaving residue on the sample (see Figure 13a). To prevent this, applying natural oils or waxes, such as coconut oil or beeswax, to the surface can facilitate the removal of the sheets (see Figure 13b).
4. Drying on plastic yields a very smooth and uniform finish, which is advantageous for material applications. However, as with wood, the samples may slightly adhere to the plastic. To address this issue, natural oils or waxes are applied to prevent sticking, Figure 14a.
5. Drying on Fabric: Unlike other methods, drying on fabric allows for easy removal of the material while achieving a smooth texture. The fabric does not wrinkle, and the sample does not take on any texture, as was the case with parchment paper. Additionally, the material remains flat without curling or wrinkling Figure 14.
6. Drying on Molds: Thanks to methods such as 3D printing or laser cutting/engraving, and the bacterial cellulose’s ability to reproduce surface details, almost any pattern can be achieved on the material during drying, as shown in Figure 15.

**Figure 16:**
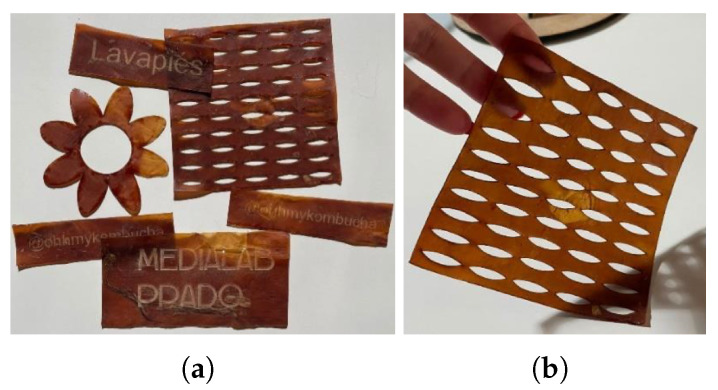
(**a**) Images of the various laser cutting and engraving tests performed. (**b**) A detail of one of the cut sheets.

Lastly, it is worth highlighting laser cutting and engraving. Although bacterial cellulose can be cut with simple tools like scissors or a utility knife, using laser cutting offers much greater precision and eliminates human error. This method allows for creating tightly fitting pieces from a single sheet, minimizing material waste. Additionally, laser engraving enables the addition of logos, names, and images, enhancing its appeal for branding and various business applications, Figure 16.

The laser cutting machine used in the experiment is a Gesmain CO2 Laser Cutter 3050, featuring a working area of 500 × 300 mm, a power output of 60 W, and a cutting speed ranging from 1 to 60 mm/s. It supports PLT, DXF, BMP, AI, and DST file formats. This cutter is capable of working with various materials and, following this experiment, it has been confirmed that it can also cut and engrave bacterial cellulose. The cutting and engraving parameters are shown in the Table 4.

#### 3.3.3. Post-Treatment

Several treatments can enhance the properties of bacterial cellulose while maintaining its biodegradability. Among these, applying a mixture of coconut oil and beeswax stands out for providing increased resistance, elasticity, and improved surface finish, as well as partial water resistance. Once again, the use of beeswax should be evaluated if a vegan product is desired.

**Figure 17:**
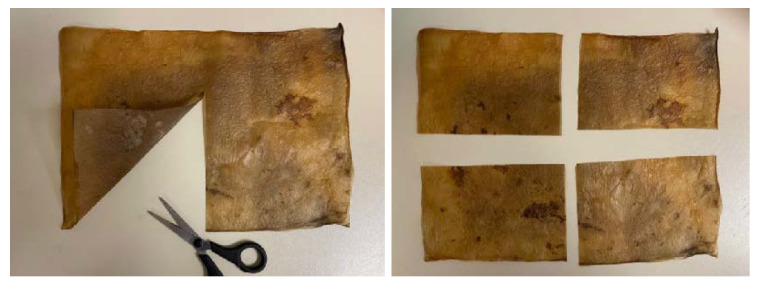
Obtaining samples for the application of the different treatments.

**Figure 18:**
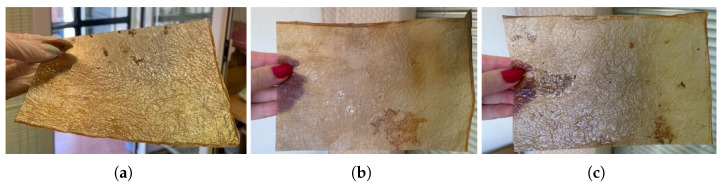
Images of the finish of the samples treated with (**a**) coconut oil, (**b**) beeswax, and (**c**) a 50% mixture of both.

To assess the impact of these treatments, a BC sample was divided into four equal parts, Figure 17. One part was treated with coconut oil, another with beeswax, a third with a mixture of both, and the fourth was left untreated Figure 18.

The coconut oil and beeswax mixture was prepared with a 50% proportion of each, using a double boiler, resulting in a homogeneous and solid paste after around half an hour of drying.

The tests revealed that coconut oil imparts an attractive shine and slight flexibility to the material, though it may leave a greasy finish if over-applied. Beeswax significantly improves water resistance compared to the mild protection provided by coconut oil alone.

In conclusion, applying a thin layer of the coconut oil and beeswax mixture to BC samples is recommended for textile applications, as it balances water resistance with esthetic properties.

#### 3.3.4. Bonding

A key aspect of eco-design and sustainability is to use a single material in product development to facilitate recycling. In this regard, experiments will be conducted on joining BC samples, utilizing their ability to bond during drying. However, since achieving a complete bond is not always feasible, sewing the samples with cellulose threads will also be explored as a viable alternative for joining the samples and supporting their integration into sustainable product design.

**Figure 19:**
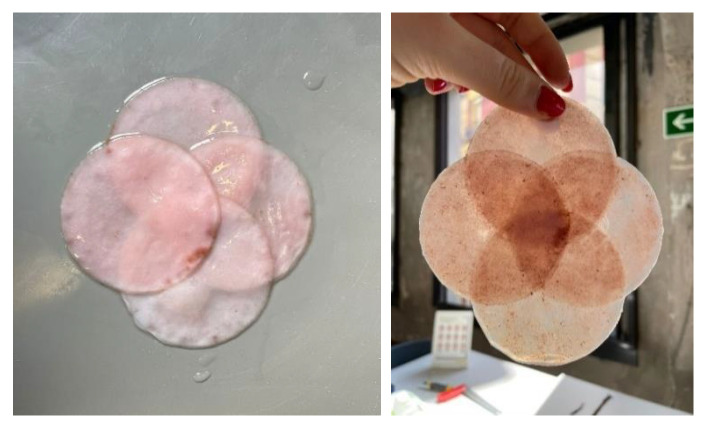
Images of the process of wet mono-material bonding and its appearance after drying.

**Figure 20:**
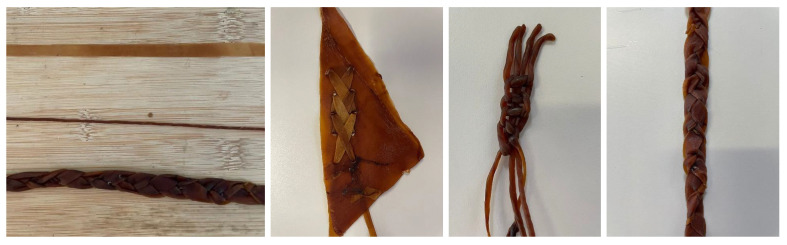
Images of the thread production process and a comparison from strips to braided threads. Examples of the potential uses of threads and strips.

1. Mono-material: To evaluate this bonding method, four different bacterial cellulose samples will be combined to form a pattern. Four samples grown in Petri dishes will be joined and then rehydrated. After one day, the samples will have dried and bonded. This approach allows for complex unions. Initial bonds are made, the material is allowed to dry, and then it is rehydrated to continue joining additional parts. This method enables the creation of more complex geometries or products with multiple sealed edges, Figure 19.
2. Stitching: As previously mentioned, bacterial cellulose can be shaped into threads, opening up a wide range of possibilities for creating forms that could not be achieved with mono-material joints or with the material in its dry state. It is important to note that the cellulose threads developed during the experimental process differ from conventional sewing threads. These threads are produced from flat strips of BC, with a diameter of approximately 1 to 1.5 mm, Figure 20.
  To study the connections of cellulose using threads and strips of the same material, the process has been approached as if it were leather, exploring methods and tools used in the leather industry. The process of making threads begins with cutting the material into strips, which can be done with scissors or laser cutting. The strips are then carefully rolled to form a cylindrical shape, resulting in thick threads. From these threads, braids can also be created, expanding the options for designing and manufacturing products with bacterial cellulose, Figure 20.

#### 3.3.5. Demonstration of Bacterial Cellulose Applications

**Figure 21:**
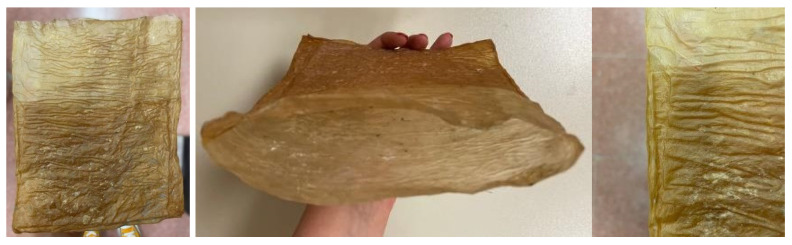
Images of the final bag and close-ups of the seam.

**Figure 22:**
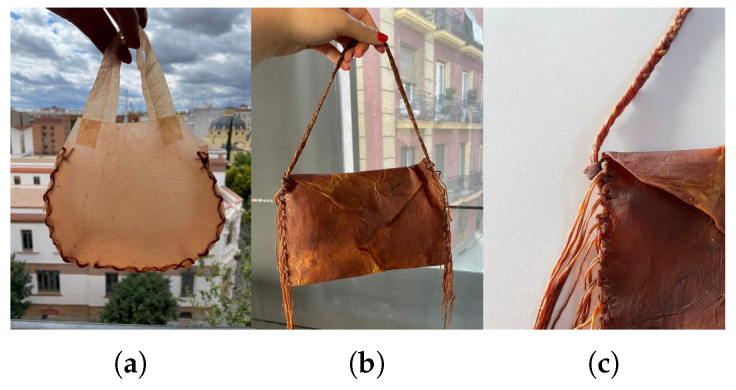
Images of the handbags: (**a**) final first handbag prototype, (**b**) images of the final prototype of the second handbag and its details in (**c**).

In light of the results obtained separately, a final demonstration was conducted by creating several designs using the techniques evaluated earlier. Specifically, a bag that could be used as packaging (Figure 21), and two handbags combining different techniques (Figure 22).

**Figure 23:**
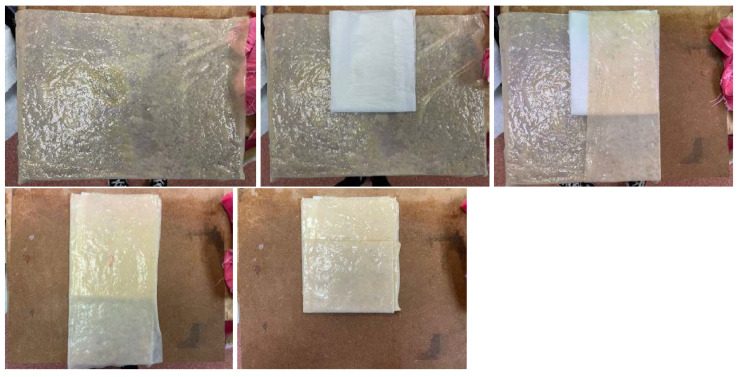
Manufacturing process of a bag by self-joining (mono-material).

In the design of the bag, only the joining of the material with itself was used. The process can be seen in Figure 23, and the final result in Figure 21.

**Figure 24:**
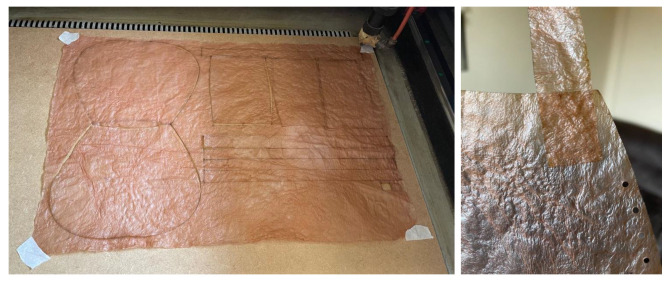
Images of the cutting and mono-material joining.

For the first handbag prototype, a BC sample dyed with beetroot and dried on fabric was used, with dimensions of 48 × 32 cm, which was cut with the previously mentioned laser. The handles of the bag were attached to the main sections using mono-material joints: the ends of the handles and the tops of the sections were moistened to ensure adhesion during drying, Figure 24.

**Figure 25:**
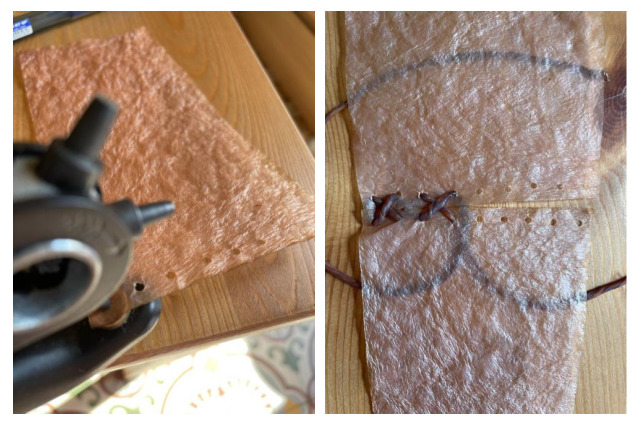
Images of the die-cutting and prototype assembly.

The joints between the main sections and the central strip of the bag were made with bacterial cellulose threads, as well as between the parts of the central section. For this, holes were punched in the ends of the sections using a hole punch. Additionally, all pieces were treated with a mixture of coconut oil and beeswax (40% coconut oil) to enhance moisture resistance and provide a glossy finish, Figure 25.

Once the joints between the handles and the main sections had dried, and the central strip was fully assembled, the final shape of the bag was formed. To achieve this, the two main sections were simultaneously attached to the central part, resulting in the final prototype shown in Figure 22.

This prototype demonstrates the successful application of the techniques studied for treating bacterial cellulose, including dyeing with natural dyes, using cellulose as threads for joints, mono-material joints, and laser cutting. The same approach was used for the second prototype shown in Figure 22.

As can be seen in the images, the material obtained resembles leather. For cleaning, a damp cloth can be used, but it is important to avoid soaking the material, as it could weaken, potentially leading to tearing or deformation. It is worth noting, as mentioned at the end of Section 3.2 “Utilization of Organic Waste”, that once the material is produced and the bonding has been completed, it undergoes a treatment to halt bacterial activity, either by boiling the sample or applying specific products. Furthermore, for textile use, it is recommended to treat the surface, as discussed in Section 3.3.3 “Post-Treatment”, with a substance that helps to waterproof the material.

## 4. Conclusions

This study on the production and application of bacterial cellulose from organic waste has highlighted the potential of this material as a sustainable alternative in the textile industry. This research has demonstrated that organic waste, such as coffee grounds and other food scraps, can be effectively utilized to produce BC, generating materials with properties comparable to those obtained using traditional methods based on tea and sugar. However, bacterial cellulose derived from organic waste exhibits greater rigidity, suggesting the need to optimize its physical properties for various applications.

A key aspect of the study was the evaluation of two cultivation methods for BC production: static and agitated. Static cultivation, the most common method for producing high-quality bacterial cellulose films, allows for the formation of sheets with an interconnected three-dimensional structure, ideal for biomedical and textile applications. However, this method presents challenges in terms of yield and extended incubation times, limiting its scalability for industrial production.

In contrast, agitated cultivation accelerates BC production by generating material in the form of pellets or aggregates. This method, by keeping the cells in constant motion, enables faster production, though with less fiber cohesion and the need for additional steps to produce sheets suitable for textile applications. Additionally, the use of starter media derived from previous fermentations has been shown to offer a more sustainable alternative, reducing waste and improving process efficiency.

**Figure 26:**
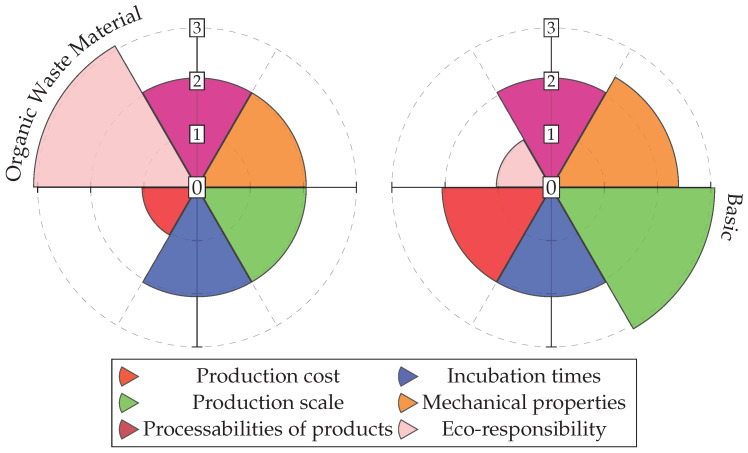
Comparison of the performance of bacterial cellulose between different BC cultures in static cultivation. Own elaboration.

Figure 26 presents a comparison between BC production using organic waste, as described in this study, and BC produced in a basic culture. Both cultures were grown under static conditions. The values are categorized into four levels: poor, medium, high or good, and very high or excellent, corresponding to a scale from 0 to 3 in the respective graphs. The graph highlights that the effort to achieve a sustainable material within a circular economy framework tends to reduce the automation of the production process and, therefore, its potential scalability. The mechanical properties and production costs are significantly higher in the basic culture, in both cases due to the greater purity of the culture medium.

The material’s ability to be dyed with natural dyes and its compatibility with treatments such as coconut oil and beeswax expand its range of applications. These treatments not only enhance the material’s esthetics and functionality but also preserve its biodegradable nature, aligning with sustainability principles.

However, it is crucial to recognize that the use of certain treatments, particularly those involving animal-derived products like beeswax or cochineal dye, could compromise the vegan status of bacterial cellulose products. As bacterial cellulose itself is a plant-based, cruelty-free material, maintaining its vegan integrity requires careful selection of all additives and treatments used in the production process. By choosing plant-based alternatives to animal-derived additives, it is possible to produce a material that meets both the ethical demands of veganism and the sustainability goals of reducing environmental impact.

The potential for laser cutting and engraving on bacterial cellulose, along with the reuse of material scraps, reinforces the concept of a circular economy and minimizes waste in the production process. The continued development of vegan-friendly treatments and dyes will further enhance the appeal of bacterial cellulose as a versatile, sustainable, and ethically responsible material, suitable for a wide range of industrial applications.

Despite the progress made, several key areas for future research have been identified. It is essential to continue optimizing bacterial cellulose production methods to improve uniformity and reproducibility, thereby facilitating its commercial viability. Evaluating scalability in industrial contexts and waste management is crucial, along with a thorough characterization of the material in terms of mechanical, optical, and chemical properties.

Additionally, improving water resistance and durability of the material should be explored, as well as investigating innovative manufacturing techniques such as 3D printing. Assessing the material’s life cycle and its sustainability impact is also vital. Finally, considering the social and community impact of biomass-based materials may promote the integration of sustainable solutions in urban environments.

## Figures and Tables

**Table 1:** Comparison of recipes for making BC. All quantities are referred to per litre of water. Fermented liquid is that obtained from a previous fermentation.

Ingredients	Schramm and Hestrim [11]	Basic [17]	This Work
Carbon source	Glucose: 20 g	Sugar: 100 g	Organic remains: 300 g
Nitrogen source	Pectone: 5 g	Tea: 3 g	Coffee/tea grounds ^1^
Microorganisms	Yeast extract: 5 g	SCOBY: 30 g	Fermented liquid: 30 g
Acidity	NaH_2_PO_4_: 2.7 g + HNO_3_: 1.5 g	Vinegar: 0.15 mL	Fermented liquid or vinegar

^1^ The quantity depends on the type of organic waste being used, but it will never exceed 5 g.

**Table 2:** Comparison of the ranges of mechanical properties of BC depending on the cultivation method.

Cultivation Method	Tensile Strength [MPa]	Elastic Modulus [MPa]	Elongation [%]
Basic, static	22.1–24.8	170–210	1.6–2.1
Basic, agitated	19.3–21.6	150–170	1.5–1.7
Basic, static, fermented liquid.	22.9–24.2	170–200	1.8–2.2
Agitated, fermented liquid.	20.0–22.2	150–170	1.6–1.8

**Table 3:** Comparison of the ranges of mechanical properties of BC depending on the feedstock.

Feedstock	Tensile Strength [MPa]	Elastic Modulus [MPa]	Elongation [%]
Basic	22.1–24.8	170–210	1.6–2.1
Mixture of organic waste (fruits and vegetables)	20.3–22.7	140–150	1.2–1.4

**Table 4:** Laser cutting and engraving parameters for BC sheets with a thickness of less than 1 mm.

Process	Speed [mm/s]	Power [%]	Corner Power [%]
Cutting	80	20	15
Engraving	150	15	-

## Data Availability

The original contributions presented in the study are included in the article, further inquiries can be directed to the corresponding author.

## Abbreviations

The following abbreviations are used in this manuscript:

BC	Bacterial cellulose
DIY	Do It Yourself
H-S	Schramm and Hestrim cultivation medium
SCOBY	Symbiotic Culture of Bacteria and Yeast
